# Effect of different flooring types on pressure distribution under the bovine claw – an ex vivo study

**DOI:** 10.1186/s12917-018-1579-9

**Published:** 2018-08-31

**Authors:** Benjamin Oehme, S. M. Geiger, S. Grund, K. Hainke, J. Munzel, C. K. W. Mülling

**Affiliations:** 1 Institute of Veterinary Anatomy, Histology and Embryology, Faculty of Veterinary Medicine, Leipzig University, Leipzig, Germany; 2 Institute of Topographic Anatomy, University of Veterinary Medicine Vienna, Vienna, Austria

**Keywords:** Bovine claw, Dairy cattle, Pressure distribution, Concrete floor, Rubber mat

## Abstract

**Background:**

Mechanical interactions between hard floorings and the sole of bovine claws can be reasonable to cause traumatic claw lesions. In this ex vivo study, the direct kinetic impact of concrete and three types of rubber mats on the sole of dairy cattle claws was analyzed. In order to apply uniform loads, isolated distal hind limbs of adult Holstein Friesian dairy cows were functionally trimmed according to the Dutch method and attached to a load applicator. Kinetic data were recorded using a thin, foil-based pressure measurement system (Hoof™ System, Tekscan®).

**Results:**

On concrete, the load distribution between the lateral and medial claw was less balanced than on the rubber floorings. The loaded area was significantly smaller on concrete (32.2 cm^2^) compared to all rubber mats (48.3–58.0 cm^2^). Average pressures (**P**_**av**_) and maximum pressures (**P**_**max**_) were significantly higher on concrete (P_av_ 44.7 N/cm^2^; P_max_ 130.3 N/cm^2^) compared to the rubber floorings (P_av_ 24.9–29.7 N/cm^2^; P_max_ 71.9–87.2 N/cm^2^). Pressure peaks occurred mainly in plantar and abaxial parts of the lateral claw and in apical and plantar regions of the medial claw. Load distribution displayed a widely unloaded slope region, but considering the pressure distribution under the claw, none of the zones showed a generally lower pressure exposure.

**Conclusions:**

Altogether, rubber floorings lead to a significant mechanical relief of the sole compared to concrete. Furthermore, relevant differences between the tested rubber mats could be determined. Therefore the used system may be applied to compare further flooring types.

## Background

Hard flooring systems such as concrete are the reason for a significantly higher risk of claw diseases and developing lameness in dairy cattle [1, 2]. Still, some studies proved a higher incidence of sole ulcers on rubber floorings than on concrete [3, 4], albeit with lower severity [5]. In contrast, Benz [6] found out that cows kept on rubber floorings showed a significantly lower incidence of claw diseases which increased again when the animals returned to concrete flooring. Besides, cows kept on rubber showed significantly more comfort behavior than those kept on concrete and walked longer distances, almost comparable to cows on pasture [7, 8].

To understand the underlying reasons of the mentioned differences in claw health and cow comfort, the effects of different floorings on the pressure load of bovine claws have been investigated in previous studies [9–11]. As most of the conducted surveys used stationary force or pressure measuring plates with hard surfaces, no direct interactions between flooring type and claw as well as the effects on the individual claw could be captured [12].

Franck et al. [12] applied thin, foil-based pressure sensitive sensors to investigate the direct impact of different concrete surface roughnesses on the pressure distribution under isolated bovine claws. With same type sensors, Nilsson et al. [13] analyzed the pressure distribution under isolated bovine distal limbs on slatted concrete flooring at different positions to the slats. In vivo measurements with large animals using these piezoresistive sensor foils have solely been performed in equine kinetic research so far [14–18].

An evaluation of these equine sensors (Tekscan® Hoof™System) found less accuracy in measuring vertical Ground Reaction Force (**vGRF**) compared to force plates, but overall results did not differ significantly [19]. Especially after proper calibration the piezoelectric sensors showed a reliable accuracy [20, 21]. The system was tested and recommended for scientific and clinical application by Lange et al. [17].

The aim of this study was to establish a laboratory setup for kinetic examinations of the bovine distal limb and to directly analyze the kinetic influence of different flooring types to the sole of dairy cattle’s claws for the first time.

## Methods

### Material

Twelve left hind limbs of adult Holstein Friesian dairy cows (age 5.0 ± 1.6 years) from an abattoir were separated below the tarsometatarsal joint immediately after slaughter and transported to the lab. All claws were trimmed according to the Dutch method [22]. The limbs and claws showed no macroscopically visible pathologies. Metatarsi were cut with a band saw in the proximal third. The medullary cavity of each metatarsus was filled with Demotec®95 (Demotec Demel e.K., Nidderau, Germany) in order to fix a threaded bolt in the medullary canal to attach the limb to the load applicator.

Four different floorings were evaluated including concrete (**con**) and three types of rubber mats (Table 1): KARERA (**Kar**), KURA (**Kur**) and profiKURA (**proK**) (Gummiwerk KRAIBURG GmbH&Co. KG, Waldkraiburg, Germany).

**Table 1:**
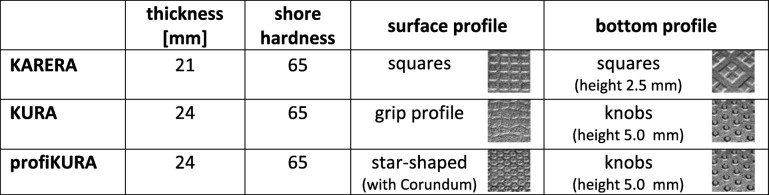
Overview and characteristics of tested rubber mats (Gummiwerk KRAIBURG GmbH&Co. KG)

### Pressure measuring system

A foil-based piezoresistive pressure measurement system (M3200E, Hoof™System, Tekscan Inc., Boston, MA, USA) was used. The sensor foils were 0.23 mm thick with a sensor matrix of 167.6 × 167.6 mm and a resolution of 3.9 sensels/cm^2^. A 1.5 mm adhesive protective foil covered the sensors on both sides.

### Experimental setup

**Fig. 1:**
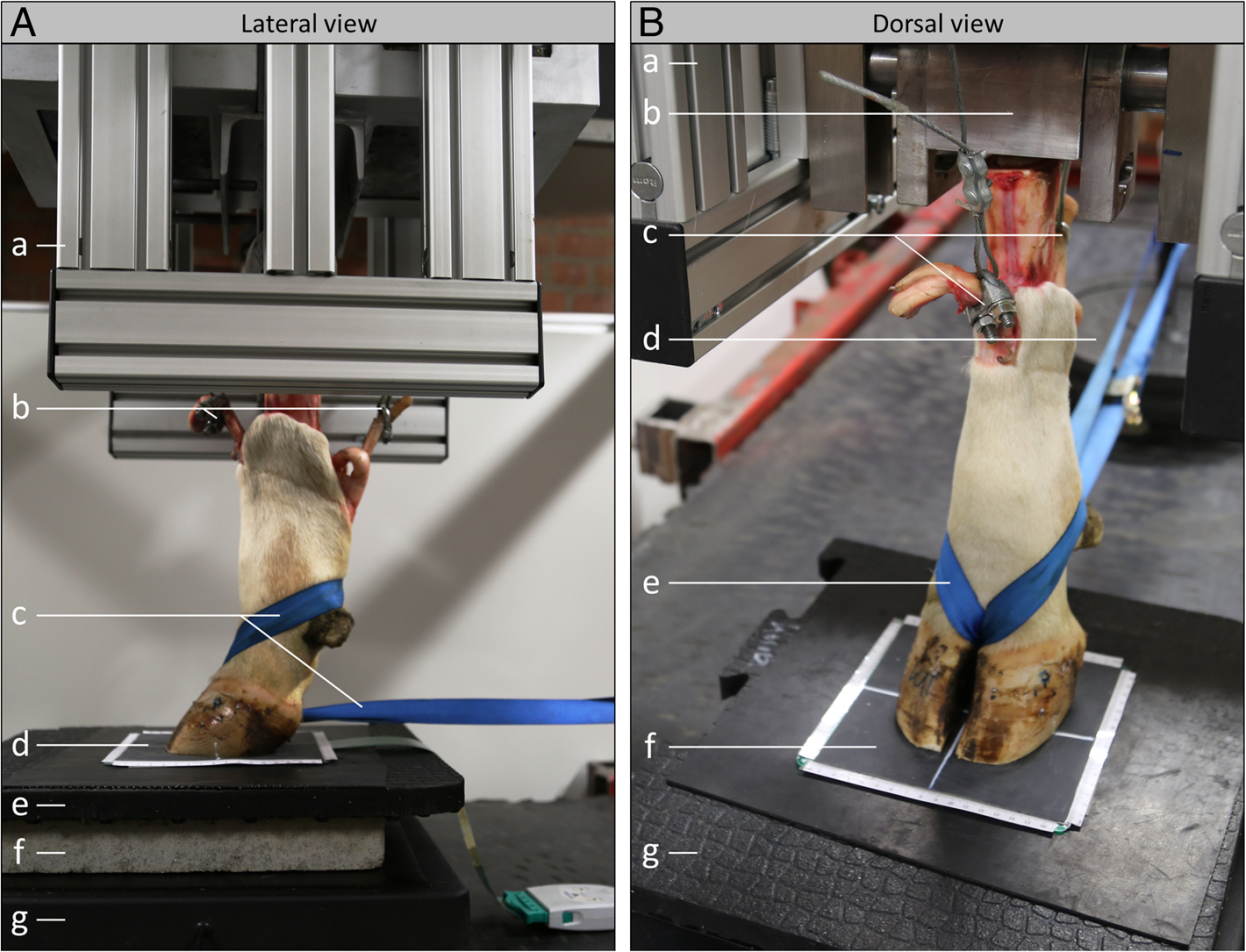
Experimental setup. **A** Lateral view: a = load applicator, b = clamped tendons, c = securing strap, d = sensor foil, e = rubber mat, f = concrete, g = digital scale. **B** Dorsal view: a = load applicator, b = cam, c = clamped tendons, d = tendon weight, e = securing strap, f = sensor foil, g = rubber mat

The distal limbs were attached to a load applicator and positioned centrally on the sensor with the metatarsus perpendicular to the tested floor. In order to simulate the in vivo situation as closely as possible the deep digital flexor tendon and the digital extensor tendons were pulled proximally with 500 and 50 N respectively. A securing strap was attached round the pastern joint to stabilize the phalangeal bone axis, which was monitored by latero-medial fluoroscopy. The limbs were loaded vertically with 240 kg resulting in a final load of 143 ± 9 kg under the claws, which was determined by a digital scale (Fig. 1). The lower resultant load was caused by the tendon traction in opposite direction and the horizontal Ground Reaction Forces, which were not captured by the sensors or the scale.

On every flooring a sequence of four repeated measurements was performed. The sensors were initially conditioned with loads up to 300 kg and subsequently calibrated before and after every sequence with 20 and 80% of the maximum applied load [23].

### Data processing

**Fig. 2:**
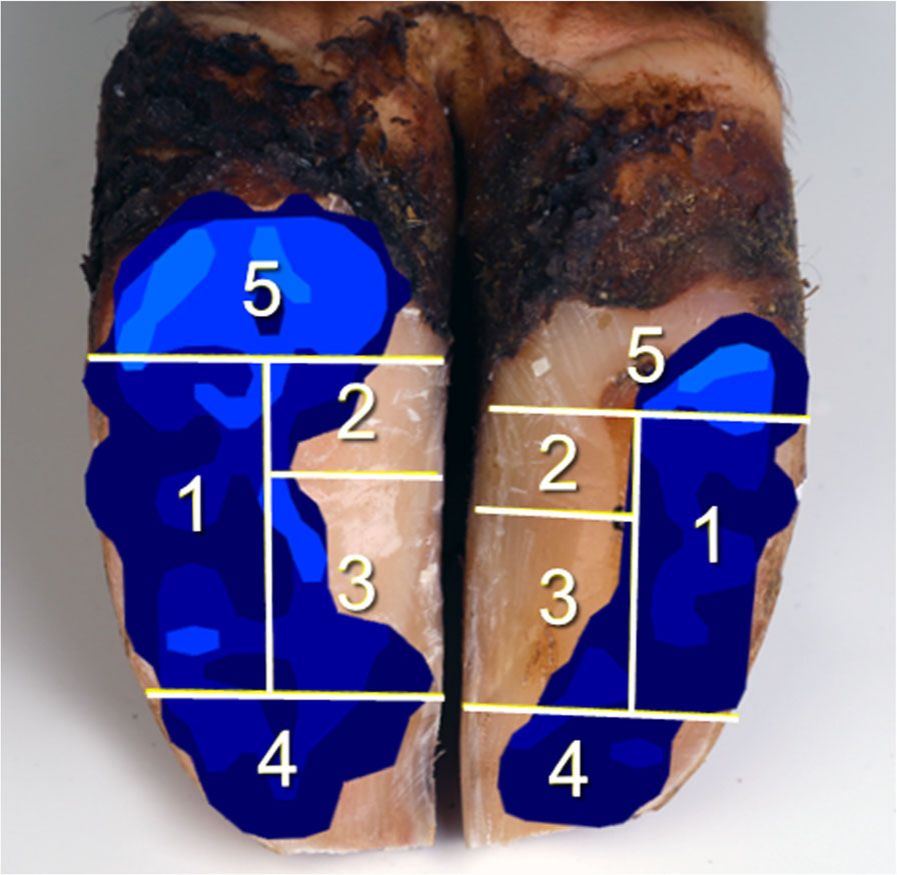
Subdivision of the sole according to Carvalho et al. [25]

In the subsequent data processing, the HoofSCAN Research software (version 6.85, Tekscan Inc., Boston, MA, USA) was used to analyze the force balance (**FB**), calculated from vertical Ground Reaction Forces (**vGRF**) of lateral and medial claw $\left( FB=\frac{vGRF_{lat}}{vGRF_{med}}\ \right)$, the total loaded area (**A**_**total**_), average pressure (**P**_**av**_) and maximum pressure (**P**_**max**_) on the four tested floorings under both claws. Afterwards, data were exported to RStudio [24] and by combining the pressure maps with computed tomography-based sole surface areas, a zonal subdivision was implemented according to Carvalho et al. [25] (Fig. 2). By this, the occurrence of P_max_ could be localized per claw. Parameters to compare the zones of each claw between the different floorings were the vGRF relative to the total vGRF per side (**F**_**side**_), the loaded area per zone relative to the total zone area (**A**_**zone**_) and the vGRF per loaded area per zone, which describes the pressure in each zone (**P**_**zone**_).

### Statistical analysis

Statistical analysis was conducted with the software RStudio [24]. Graphical assessment and the Shapiro-Wilk test were applied to determine normal distribution. As numerous data frames were non-parametric, statistical significances were tested using Wilcoxon signed-rank test. Post hoc correction was carried out with Bonferroni-Holm procedure. First of all, differences of overall parameter data (A_total_, P_av_, P_max_) were analyzed between the four tested floors. Subsequently, parameters regarding the zones were examined separately for the lateral and medial claw. The parameters A_zone_ and P_zone_ were compared between the tested floorings for every zone. F_side_ was analyzed for differences between the floorings and between the five zones. Differences were considered to be significant at *p* < 0.05.

## Results

For all tested floorings, the overall load was distributed unevenly between the lateral and medial claw on average. On concrete, the lateral claw was loaded 4.2 times higher than the medial claw. This imbalance decreased significantly on the tested rubber floorings, where the load on the lateral claw was 3.4 (Kar), 2.8 (Kur) and 2.7 (proK) times higher compared to the medial claw.

**Fig. 3:**
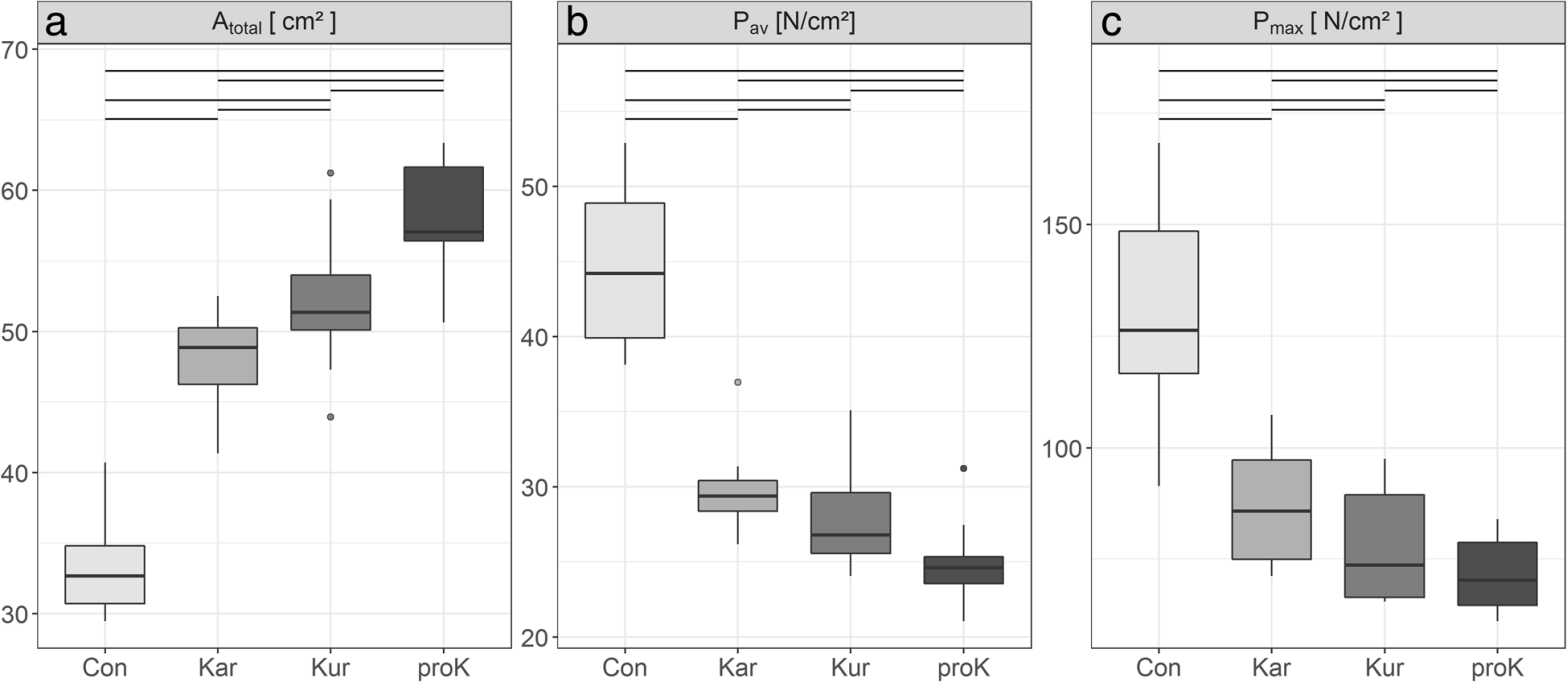
**a** Contact area, **b** average pressure and **c** maximum pressure. Boxplots of values on the four tested floorings (concrete (con) and the three rubber mats KARERA (Kar), KURA (Kur) and profiKURA (proK)). Horizontal lines at the top indicate significant differences between the flooring types

**Fig. 4:**
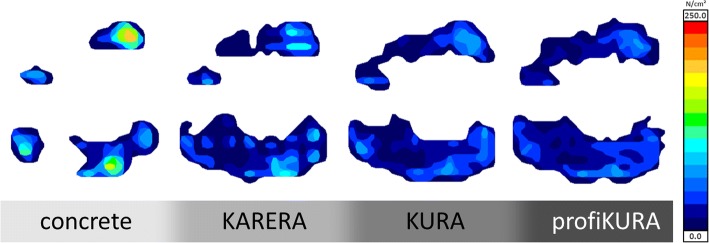
Exemplary pressure imprints of one claw on the different tested floorings

The total loaded area (Fig. 3a) was significantly smaller on concrete (33.2 ± 3.4 cm^2^) than on all tested rubber floorings and differed significantly between all types of rubber mats (Kar 48.3 ± 3.5 cm^2^, Kur 52.3 ± 4.8 cm^2^, proK 58.0 ± 3.8 cm^2^) (Fig. 4).

The lowest average pressure (Fig. 3b) was found on profiKURA (24.9 ± 2.6 N/cm^2^). Average pressure differed significantly between all floorings and was highest on concrete (Kur 27.6 ± 3.3 N/cm^2^, Kar 29.7 ± 2.7 N/cm^2^, con 44.7 ± 5.5 N/cm^2^).

Also maximum pressure values (Fig. 3c) were significantly higher on concrete (130.3 ± 23.7 N/cm^2^) compared to all rubber floorings. Significant differences occurred between the three types of rubber flooring as well (Kar 87.2 ± 13.4 cm^2^, Kur 78.6 ± 12.6 cm^2^, proK 71.9 ± 8.3 cm^2^).

**Fig. 5:**
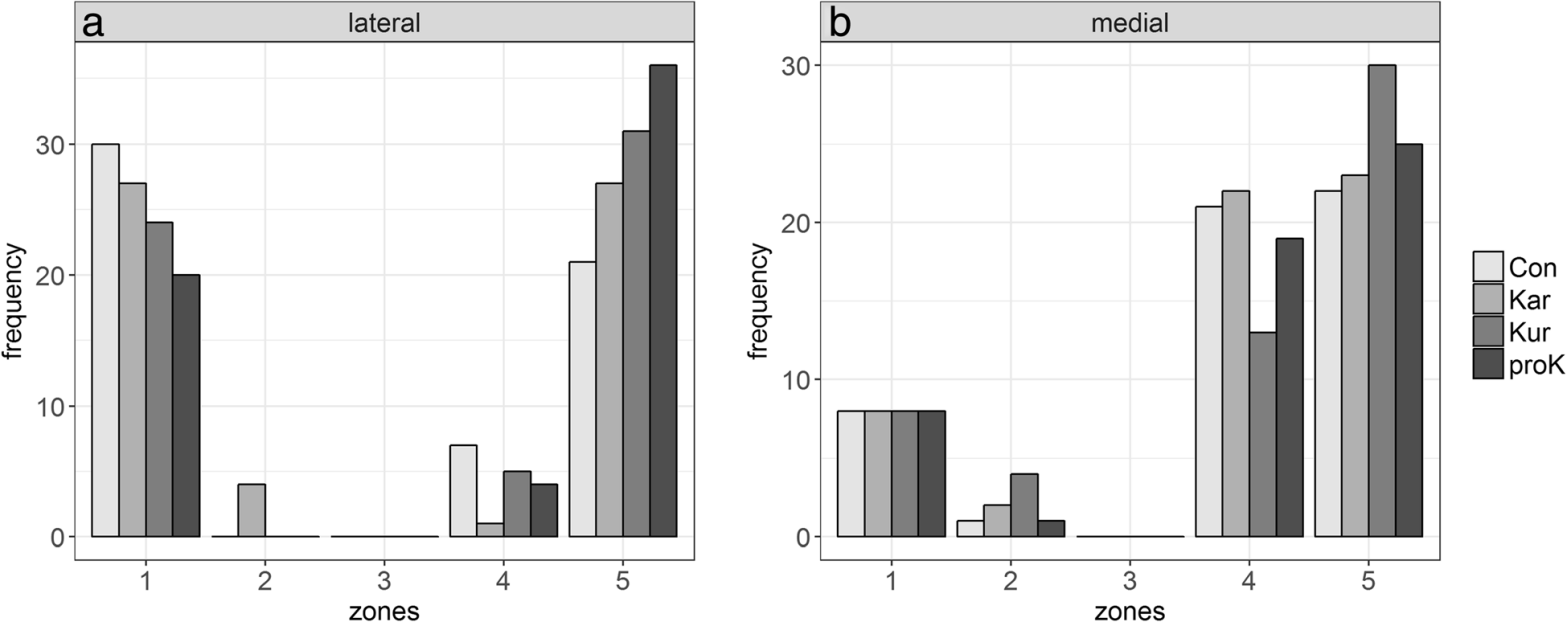
Localization of maximum pressure peaks for the **a** lateral and **b** medial claw

Analysis of kinetic characteristics in the different zones showed the following results: The majority of maximum pressure values in the lateral claw were measured in zones 1 and 5 and rarely in zone 4. Additionally, the occurrence of maximum pressures decreased in zone 1 and increased in zone 5 depending on type of flooring (Fig. 5). Considering the medial claw, the maximum pressures appeared mainly in zones 4 and 5 and to some extent in zone 1. In both claws, isolated pressure peaks occurred in zone 2 and none in zone 3.

There were no significant differences in F_side_ between the different floorings. Considering F_side_ averaged over all tested floors, zones 1 and 5 bore the highest vertical Ground Reaction Forces (vGRF) in the lateral claw (F_side_ mean: 40.1 and 32.1%). In the medial claw the highest amount of load appeared in zones 1, 4 and 5 (F_side_ mean: 24.8, 35.2 and 28.3%). Zones 2 and 3 carried the smallest loads on both sides (F_side_ between 3.4 and 8.6%) and were significantly different in terms of the vGRF to all other zones. Furthermore, significant differences could be determined between zone 1 and 4 as well as 4 and 5 in the lateral claw (Table 2).

**Table 2:** Means and standard deviations of F_side_

Zone	1	2	3	4	5
Lateral claw					
all floorings	0.40 ± 0.12^2,3,4^	0.08 ± 0.03^1,4,5^	0.07 ± 0.04^1,4,5^	0.13 ± 0.05^1,5^	0.32 ± 0.10^2,3,4^
Medial claw					
all floorings	0.25 ± 0.16^2,3^	0.05 ± 0.04^1,4,5^	0.06 ± 0.08^1,4,5^	0.35 ± 0.24^2,3^	0.28 ± 0.14^2,3^

Data averaged over all tested flooring types for the lateral and medial claw and all five zones. Superscript numbers indicate significant differences between the stated zones

**Fig. 6:**
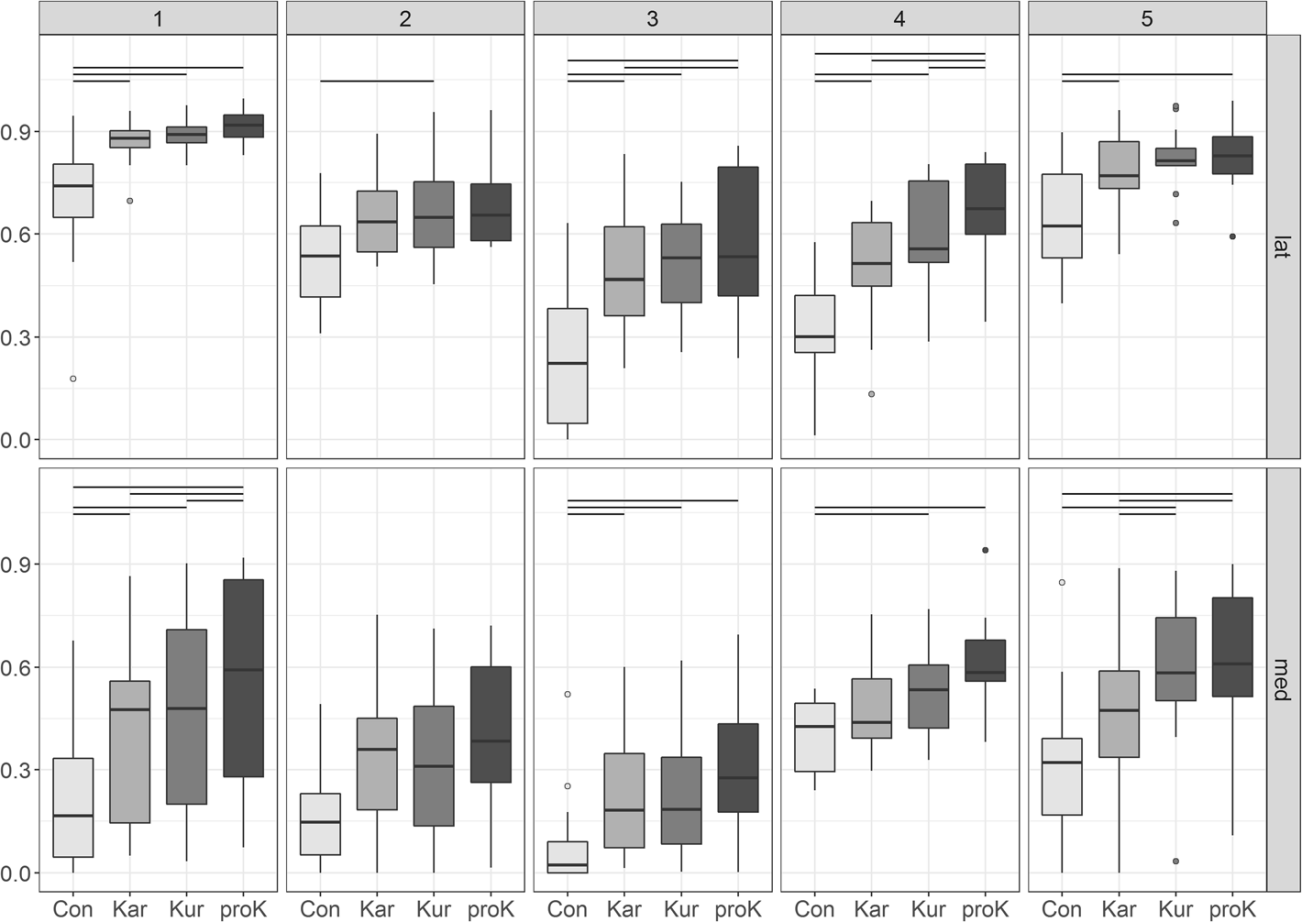
Loaded area per zone relative to total zone area. Boxplots of A_zone_ for the lateral and the medial claw, all five zones and the four tested floorings (concrete (con) and the three rubber mats KARERA (Kar), KURA (Kur) and profiKURA (proK)). Horizontal lines at the top indicate significant differences between the flooring types

Generally, the relative loaded area per zone (A_zone_) increased on rubber mats compared to concrete (Fig. 6). Except for zone 2 the loaded area differed significantly between concrete and at least two rubber mats. There were also some significant differences in loaded areas between the tested rubber floorings (Table 3).

**Table 3:** Means and standard deviations of A_zone_

Zone	1	2	3	4	5
Lateral claw					
con	0.69 ± 0.21 ^Kar,Kur,proK^	0.53 ± 0.15 ^Kur^	0.24 ± 0.21^Kar,Kur,proK^	0.33 ± 0.16 ^Kar,Kur,proK^	0.65 ± 0.17 ^Kar,proK^
Kar	0.87 ± 0.07 ^con^	0.13 ± 0.60	0.49 ± 0.20 ^con,proK^	0.50 ± 0.17 ^con,proK^	0.78 ± 0.12 ^con^
Kur	0.89 ± 0.05 ^con^	0.16 ± 0.05 ^con^	0.52 ± 0.16 ^con^	0.58 ± 0.17 ^con,proK^	0.82 ± 0.09
proK	0.91 ± 0.05 ^con^	0.13 ± 0.06	0.58 ± 0.20 ^con,Kar^	0.66 ± 0.17 ^con,Kar,Kur^	0.83 ± 0.11 ^con^
Medial claw					
con	0.21 ± 0.20 ^Kar,Kur,proK^	0.16 ± 0.14	0.09 ± 0.16 ^Kar,Kur,proK^	0.40 ± 0.11 ^Kur,proK^	0.31 ± 0.25 ^Kur,proK^
Kar	0.42 ± 0.26 ^con,proK^	0.33 ± 0.22	0.22 ± 0.18 ^con^	0.49 ± 0.15	0.48 ± 0.25 ^Kur,proK^
Kur	0.48 ± 0.29 ^con,proK^	0.33 ± 0.22	0.24 ± 0.22 ^con^	0.54 ± 0.15 ^con^	0.58 ± 0.23 ^con,Kar^
proK	0.57 ± 0.30 ^con,Kar,Kur^	0.40 ± 0.23	0.32 ± 0.23 ^con^	0.61 ± 0.15 ^con^	0.63 ± 0.22 ^con,Kar^

Relative values for the lateral and the medial claw, all five zones and the four tested floorings (concrete (con) and the three rubber mats KARERA (Kar), KURA (Kur) and profiKURA (proK)). Superscript abbreviations indicate significant differences between these floorings

**Fig. 7:**
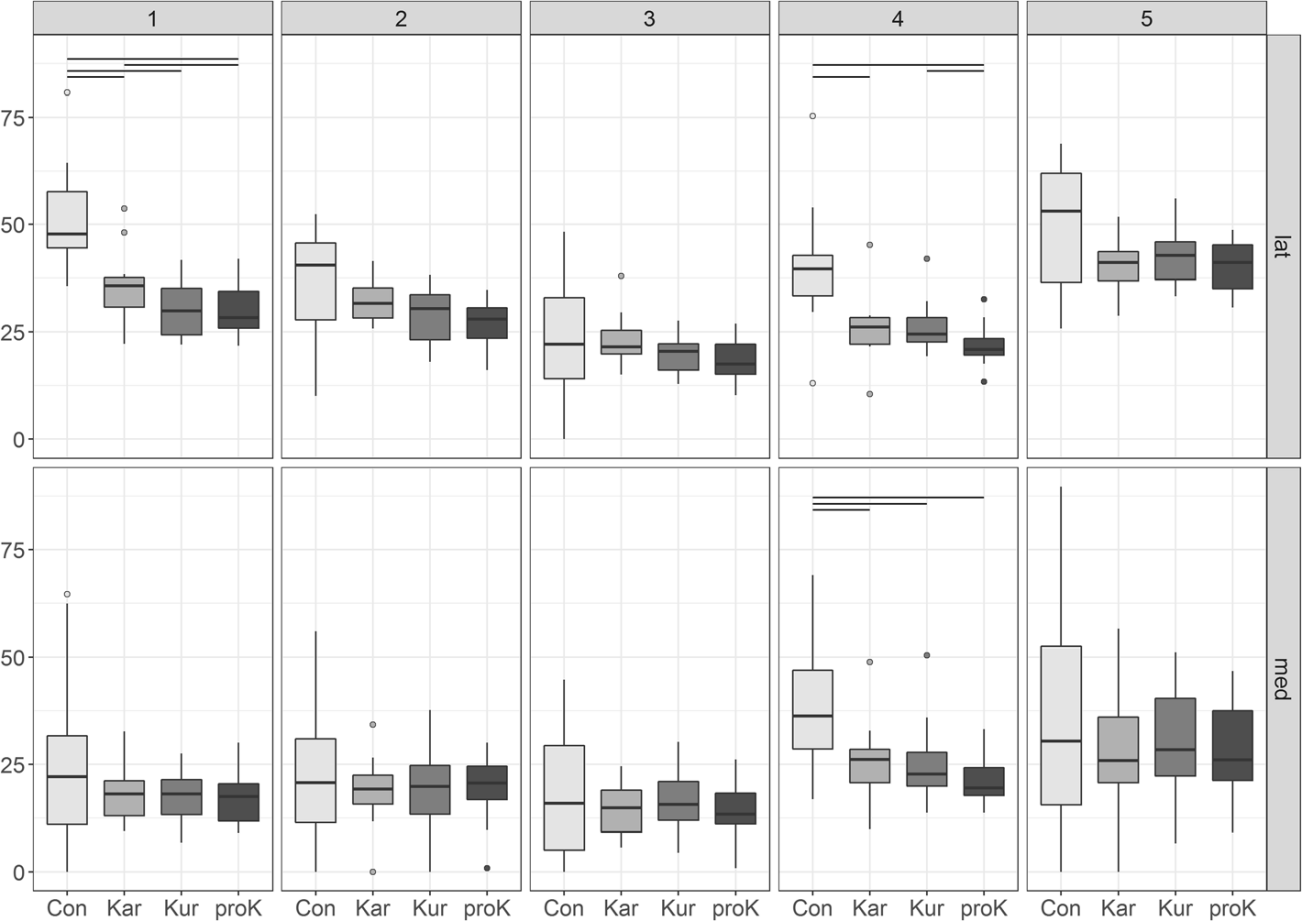
Pressure in each zone, referred to the loaded area. Boxplots of P_zone_ [N/cm^2^] for the lateral and the medial claw, all five zones and the four tested floorings (concrete (con) and the three rubber mats KARERA (Kar), KURA (Kur) and profiKURA (proK)). Horizontal lines at the top indicate significant differences between the flooring types

The pressure in each zone based on the loaded area (P_zone_) showed significant differences between concrete and rubber floorings for the lateral zones 1 and 4 and for the medial zone 4 (Fig. 7). In the loaded areas of the slope, represented by zones 2 and 3, pressure loads (14.52 N/cm^2^–37.05 N/cm^2^) similar to zones 1, 4 and 5 (17.19 N/cm^2^–50.49 N/cm^2^) occurred (Table 4).

**Table 4:** Means and standard deviations of P_zone_ [N/cm^2^]

Zone	1	2	3	4	5
Lateral claw					
con	51.25 ± 12.36 ^Kar,Kur,proK^	36.51 ± 12.34	22.89 ± 14.53	40.20 ± 14.17 ^Kar,proK^	50.11 ± 13.75
Kar	35.85 ± 8.24 ^con, proK^	32.33 ± 4.64	23.28 ± 5.99	26.02 ± 7.57 ^con^	40.69 ± 6.19
Kur	30.40 ± 6.05 ^con^	28.87 ± 6.49	19.98 ± 4.54	26.37 ± 5.95 ^proK^	42.81 ± 7.28
proK	29.76 ± 5.78 ^con, Kar^	26.34 ± 5.44	18.54 ± 4.82	22.07 ± 4.91 ^con,Kur^	40.35 ± 5.54
Medial claw					
con	25.22 ± 20.51	21.57 ± 15.50	18.17 ± 15.04	38.05 ± 14.81 ^Kar,Kur,proK^	37.06 ± 29.35
Kar	18.55 ± 6.53	18.78 ± 8.10	14.63 ± 5.59	25.76 ± 9.04 ^con^	27.99 ± 13.88
Kur	18.01 ± 5.95	19.34 ± 9.79	16.38 ± 7.16	25.45 ± 9.44 ^con^	29.93 ± 12.40
proK	17.24 ± 5.93	19.35 ± 7.85	13.92 ± 6.34	21.56 ± 6.31 ^con^	27.72 ± 10.96

Pressure values for the lateral and the medial claw, all five zones and the four tested floorings (concrete (con) and the three rubber mats KARERA (Kar), KURA (Kur) and profiKURA (proK)). Superscript abbreviations indicate significant differences between these floorings

## Discussion

A physiological positioning and loading of isolated limbs is hard to achieve. The load applicator used in this study was specifically designed for static load application on isolated bovine distal limbs. The applied load of 150 kg was based on findings of Chapinal et al. [26] and Fischer [27] and was found suitable to mimic the load situation in living animals. As no specific data for cattle are available, tendon loads were adapted to tendon strains in walking ponies [28] and adjusted to a feasible order of magnitude. Previous studies suppressed the influence of most parts of the limbs’ anatomical structures by fixating the flexible sections [13] or by solely using the horn capsules [29, 30] for standardized kinetic evaluation of flooring impacts on the claws. As these designs neglect physiological dynamic properties, conclusions to in vivo conditions have to be drawn carefully [29]. Therefore, the setup in the present study influenced the movement of joints and tendons as little as possible but still emphasized the normal axis of the distal phalangeal bones, which was monitored with fluoroscopy. Still, after several load applications a dorsiflexion of the pastern joint was observed in preliminary tests. Therefore this joint was stabilized with a securing strap to achieve a consistent, physiological bone axis and force transmission over all repeated measurements (Fig. 1). Despite this, the experimental setup in this study emulates the in vivo situation as closely as possible and enables a standardized examination of effects of different flooring types by exclusion of most interfering and environmental impacts such as body weight, motion, load shifting etc.

The Tekscan® Hoof™System was applied to bovine kinetic research for the first time in this study. It has frequently been used in equine research and was recommended for scientific and clinical application by Lange et al. [17]. As some authors stated a lack of accuracy in using this piezoresistive foil sensors [19, 21, 31], most equine studies were confined to relative comparisons [14, 15, 17]. However, by use of an appropriate calibration under trial conditions and expected loads, an adequate accuracy could be obtained with this sensors [19–21]. Therefore, the sensors in this study were calibrated frequently and under measurement conditions to minimize the inaccuracy and to guarantee the same conditions for all measurements [23].

The load imbalance between the lateral and medial claw is in accordance with previous in vivo studies [32–34]. However, the lateral claw carried an even higher part of the total load in this study. This may be due to the strict perpendicular position of the limb and the lack of compensatory movement of the proximal structures. Therefore, a larger part of the force is transmitted to the longer lateral digit [35]. The reduction of imbalance on KURA and profiKURA compared to concrete shows a balancing effect of these rubber floorings on load distribution. Furthermore, the loaded area showed significant differences between the three tested rubber floorings. As all rubber mats have the same core structure and shore hardness (Table 1), the larger contact area might originate from the mats’ thickness and surface structure. KARERA is thinner compared to the other tested rubber mats and has no knobs underneath, which can explain the smaller contact areas and higher pressure loads compared to KURA and profiKURA. KURA and profiKURA only differ in surface relief and a softer, thin corundum containing top layer on profiKURA. These differences in surface texture may provide smoother contact to the sole and obviously lead to a more regular load distribution.

The higher average and maximum pressures on concrete compared to the rubber floorings result from the smaller loaded area combined with a uniformly applied load. Especially the high pressure peaks on concrete flooring are capable of damaging the claw horn tissue, in particular in the soft bulb, which has a lower compressive strength than the wall horn [36]. As visible in Fig. 3c, these pressure peaks can be reduced considerably on the tested rubber floorings.

Additionally, the variance of pressure peaks is much higher on concrete than on the rubber floorings, which is consistent with findings of Luo et al. [37]. The high influence of the claws itself on the pressure distribution [29] may explain the generally high deviations found in the present study. Therefore it is advisable to compare the effect of different floorings and treatments rather within each claw than between entire groups of limbs.

By comparing the results of this kinetic analysis with the in vivo findings of van der Tol et al. [33] and Fischer [27], the contact area in the current study is substantially smaller. This may originate from the concrete’s irregular surface compared to the smooth metal surfaces of the pressure plates used in the former surveys. The same applies to the higher maximum pressure values occurring in the present study compared to the analyzed pressure peaks of van der Tol et al. [33]. However, the average pressure was concordant with results of Telezhenko et al. [11] and van der Tol et al. [33]. Thus, the magnitude of the pressure loads determined in this study can be transferred to in vivo conditions and the effect of flooring types on the load under live cattle’s claws can be estimated.

While maximum pressures occur particularly in the plantar and abaxial zones of the lateral claw, the medial claw bears maximum pressures both in the apical and plantar zones and fewer pressure peaks were found in the abaxial zone. This is largely in accordance with the findings of Carvalho et al. [25]. Although van der Tol et al. [38] used a zonal subdivision different from the present study, parallels in localization of maximum pressure peaks can be established. Still a few pressure peaks, mainly on rubber floorings, occurred in the plantar sectors of the slope, which represents the region prone to sole ulcer. This may explain the higher prevalence of sole ulcers in dairy cattle on rubber floorings found by some authors [3, 4]. This, in turn, may lead to the conclusion that the Dutch method [22], which was challenged by other authors before [25, 33], may not be appropriate for dairy cows housed on rubber flooring systems.

The relative distribution of vGRF on the different zones (F_zone_) showed that zones 2 and 3 carried only a little proportion of the exerted load. That implies that these zones representing the slope were less burdened compared to the other zones. However, analysis of average pressure showed no general relief of these two zones. Consequently, the impacting forces might be lower but limited to a smaller area in the zones 2 and 3, which results in a pressure extent similar to the other zones.

Although an increase of loaded area between concrete and the tested rubber floorings was observed in all zones except zone 2, no associated overall decrease of mean pressure could be determined. The lack of significant differences in pressure loads between the floorings may originate from the widely varying loading patterns between the examined distal limbs. That may also be caused by the missing compensating movement of proximal structures in the used load applicator. Further, the influence of the claws itself may be a reason for the varying load distribution patterns, which has been stated by Franck et al. [12] before. Therefore, general inferences on pressure distribution over the different regions of the claws’ contact area have to be drawn carefully and may require a greater number of tested limbs.

Nevertheless, the effects of the tested rubber floorings on the overall kinetic patterns of the claw (FB, A_total_, P_av_, P_max_) showed a clear reduction of mechanical stress to the claws’ sole in comparison to concrete. Comparable effects were recently found by Hüppler et al. [15] between concrete and soft floorings in standing and walking horses. In the present study, even differences between the tested rubber floorings in loaded area and overall pressure load could be observed. It seems, that profiKURA induces the least mechanical stress, followed by KURA and KARERA.

This investigation serves as a basis for analyzing direct kinetic influences of different flooring properties on the bovine claw in live animals. To confirm the results obtained in this study, further analysis with more specimens might be needed. Furthermore, the measuring system and the ex vivo findings have to be validated in live cows, as compensating movements of proximal structures or the location of the body’s center of gravity cannot be simulated with this setting. Nevertheless, the ex vivo setup is beneficial regarding restrictions in terms of welfare reason and financial considerations and can be used to assess effects of further floorings or claw trimming methods without applying them to live animals initially.

## Conclusion

In this study it was possible to detect lower pressure loads on rubber floorings compared to concrete. Furthermore, even differences between varying rubber floorings could be determined. This reduction of mechanical stress, in particular maximum pressure peaks, is one component to reduce the risk of mechanically induced claw diseases.
